# High expression of EPB41L5, an integral component of the Arf6-driven mesenchymal program, correlates with poor prognosis of squamous cell carcinoma of the tongue

**DOI:** 10.1186/s12964-016-0151-0

**Published:** 2016-11-21

**Authors:** Yutaro Otsuka, Hiroki Sato, Tsukasa Oikawa, Yasuhito Onodera, Jin-Min Nam, Ari Hashimoto, Kiyoshi Fukunaga, Kanako C. Hatanaka, Yutaka Hatanaka, Yoshihiro Matsuno, Satoshi Fukuda, Hisataka Sabe

**Affiliations:** 1 Department of Molecular Biology, Graduate School of Medicine, Hokkaido University, North 15, West 7, Kita-ku, Sapporo, Hokkaido 060-8638 Japan; 2 Department of Otolaryngology, Head and Neck Surgery, Graduate School of Medicine, Hokkaido University, North 15, West 7, Kita-ku, Sapporo, Hokkaido 060-8638 Japan; 3 Global Station for Quantum Medical Science and Engineering, Global Institution for Collaborative Research and Education Hokkaido University, Sapporo, Hokkaido 060-8648 Japan; 4 Department of Surgical Pathology, Hokkaido University Hospital, North 15, West 7, Kita-ku, Sapporo, Hokkaido 060-8638 Japan

**Keywords:** HNSCC, Tongue SCC, EPB41L5, Invasive activity, Chemoradiation resistance

## Abstract

**Background:**

Squamous cell carcinoma of the tongue (tongue SCC) is a major subtype of head and neck squamous cell carcinoma (HNSCC), which is an intractable cancer under current therapeutics. ARF6 and its effector AMAP1 are often overexpressed in different types of cancers, such as breast cancer and renal cancer, and in these cancers, AMAP1 binds to EPB41L5 to promote invasion, metastasis, and drug resistance. EPB41L5 is a mesenchymal-specific protein, normally induced during epithelial-mesenchymal transition (EMT) to promote focal adhesion dynamics. Similarly to breast cancer and renal cancer, the acquisition of mesenchymal phenotypes is the key process that drives the malignancy of HNSCC. We previously showed that the overexpression of AMAP1 in tongue SCC is statistically correlated with the poor outcome of patients. In this study, we examined whether tongue SCC also expresses EPB41L5 at high levels.

**Results:**

Immunohistochemical staining of clinical specimens of tongue SCC demonstrated that high expression levels of EPB41L5 statistically correlate with poor disease-free survival and poor overall survival rates of patients. The tongue SCC cell line SCC-9, which overexpress Arf6 and AMAP1, also expressed EPB41L5 at high levels to promote invasiveness, whereas the weakly invasive SCC-25 cells did not express EPB41L5 at notable levels. Among the different EMT-associated transcriptional factors, ZEB1 was previously found to be most crucial in inducing EPB41L5 in breast cancer and renal cancer. In contrast, expression levels of *ZEB1* did not correlate with the expression levels of *EPB41L5* in tongue SCC, whereas *KLF8* and *FOXO3* levels showed positive correlations with *EPB41L5* levels. Moreover, silencing of EPB41L5 only marginally improved the drug resistance of SCC-9 cells, even when coupled with ionizing radiation.

**Conclusion:**

Our results indicate that activation of the cancer mesenchymal program in tongue SCC, which leads to EPB41L5 expression, closely correlates with the poor prognosis of patients. However, ZEB1 was not the major inducer of EPB41L5 in tongue SCC, unlike in breast cancer and renal cancer. Thus, processes that trigger the mesenchymal program of tongue SCC, which drives their malignancies, seem to be substantially different from those of other cancers.

## Findings

Head and neck squamous cell carcinoma (HNSCC) is one of the most common cancers in the world [1]. Over 600,000 new cases of HNSCC are reported annually [1]. Despite the recent advancements of cancer therapeutics, over 350,000 HNSCC patients die each year [1]. Squamous cell carcinoma of the tongue (tongue SCC) is a major subtype of HNSCC, accounting for approximately 25% of all HNSCC cases [1]. In general, clinical characteristics and treatment strategy of tongue SCC are similar to those of other HNSCCs. Surgical resection is the primary choice for the treatment of HNSCC. However, many cases of HNSCC are non-operative, due to a late diagnosis with locally advanced malignancy, or surgery should be avoided to maintain the patients’ quality of life. The classical drug cisplatin is still the major drug used for the treatment of HNSCC, often coupled with radiation [2, 3]. Whereas these treatments generally exhibit cancer-reducing effects, there are many cases that show relapse, primarily due to the acquisition of resistance to such treatments.

A number of studies have indicated that the acquisition of mesenchymal properties by cancer cells is closely associated with the generation of cancer stem cell-like cells, as well as the acquisition of drug resistance [4]. Although many such studies primarily dealt with breast cancer, the acquisition of mesenchymal properties has also been highly implicated in the malignancy of HNSCC [5]. We previously showed that the small GTPase ARF6 and its effector AMAP1 are often overexpressed in different types of cancers, including breast cancer and renal cancer, and play pivotal roles in promoting invasion, metastasis, and drug resistance [6–18]. We have moreover shown that AMAP1 binds to EPB41L5 [18], which is normally induced during epithelial-mesenchymal transition (EMT) [11]. Thus, the ARF6-AMAP1-EPB41L5 pathway appears to be a cancer-specific pathway that is not expressed in cancer cells unless they undergo EMT-like changes. The ARF6 pathway promotes β1 integrin recycling and the downregulation of E-cadherin, thus enhancing cell adhesion dynamics and motile phenotypes [10, 13, 18].

**Fig. 1:**
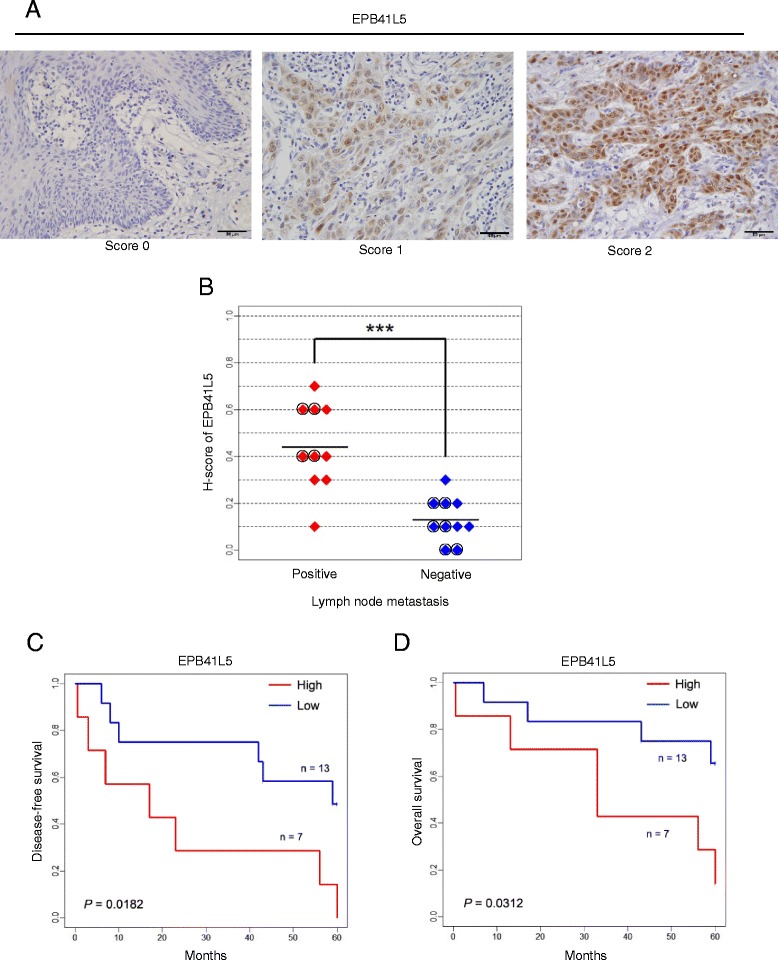
Immunohistochemical staining of EPB41L5 in primary tongue SCCs. **a** Specimens were immunohistochemically stained with polyclonal anti-EPB41L5 antibodies. Each tissue section was scored for staining intensity on a scale of 0–2, as described in Methods (H-score). A representative image for each staining is shown. Bars, 50 μm. **b** Patients were classified into two groups by the existence of lymph node metastasis. The H-score of each patient was plotted, and the *t*-test was performed. *Black circles* indicate patients without any adjuvant therapy. Black lines indicate the means. ***P < 0.001. **c** and **d** Patients with H-scores in the top 33% were classified into the “high expression” group, and the others into the “low expression” group. Kaplan-Meier curves were drawn regarding disease-free survival (**c**) and overall survival (**d**) of the patients. To evaluate the *P*-values of these analyses, the logrank test was performed

We previously reported that the AMAP1 protein is often expressed at high levels in clinical specimens of tongue SCC, and is statistically correlated with the poor prognosis of patients [15]. We here examined whether tongue SCC also express the mesenchymal component of the ARF6-based pathway, namely EPB41L5, and whether its expression correlates with malignancy. Tongue SCC patients, having been subjected to the curative resection of primary sites (Table 1), were classified into two groups based on the presence (positive) or the absence (negative) of lymph node metastasis; and EPB41L5 protein expression at the primary lesions were evaluated by the H-score (Fig. 1a).

**Table 1:** Clinicopathological characteristics of patients

Characteristic		No. of patients (%)
Sex	Male	15/20 (75)
	Female	5/20 (25)
Age (years)	30–39	3/20 (15)
	40–49	2/20 (10)
	50–59	4/20 (20)
	60–69	6/20 (30)
	70-	4/20 (20)
Location of tumor	Oral cavity (tongue)	20/20 (100)
T classification	T1	0 (0)
	T2	13/20 (65)
	T3	7/20 (35)
	T4	0 (0)
N classification	N0	10/20 (50)
	N1	7/20 (35)
	N2	3/20 (15)
M classification	M0	20/20 (100)
	M1	0/20 (0)
Differentiation	Well differentiated	12/20 (60)
	Moderately differentiated	7/20 (35)
	Poorly differentiated	1/20 (5)
Neoadjuvant therapy	Radiation therapy	7/20 (35)
	Chemotherapy	1/20 (5)
	Chemoradiation therapy	2/20 (10)
	No therapy	10/20 (50)

We found that the average H-score of the “positive” group was 0.45, whereas that of the “negative” group was 0.15 (*P* < 0.001, Fig. 1b). These results suggested a statistical correlation between the enhanced expression of EPB41L5 and the metastatic properties of tongue SCC.

We next investigated whether EPB41L5 expression levels are correlated with disease-free survival rates and overall survival rates of tongue SCC patients. The above-mentioned cohort of patients were then classified into two groups based on their expression levels of EPB41L5: patients with H-scores in the top 33% were classified into the “high expression” group, and the others into the “low expression” group (Fig. 1c, also see Methods). At 60 months after diagnosis, none of the patients of the “high expression” group were found to be disease-free, whereas 54% of the “low expression” group remained disease-free (*P* = 0.0182, Fig. 1c). Consistently, 69% of the “low expression” group survived until 60 months after diagnosis, whereas only 14% of the “high expression” group survived until that time (*P* = 0.0312, Fig. 1d). These results indicated a tight statistical correlation between high expression levels of EPB41L5 and the poor prognosis of tongue SCC patients. Thus, we propose that immunohistochemical staining of EPB41L5 provides a biomarker predictive for the prognosis of tongue SCC.

**Fig. 2:**
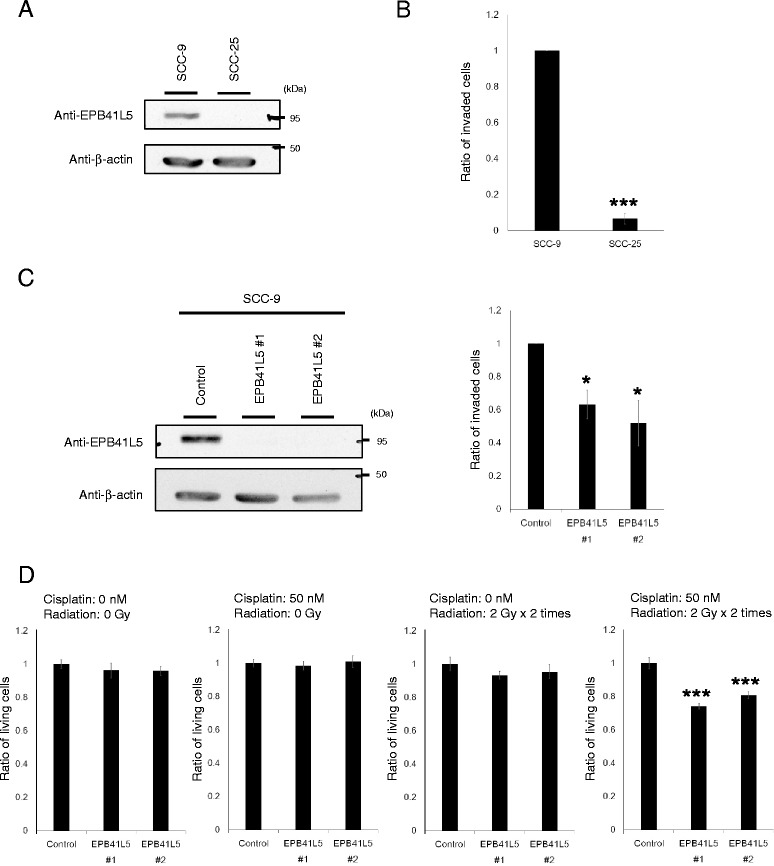
EPB41L5 promotes cell invasiveness of a tongue SCC cell line. **a** Expression of EPB41L5 in the tongue SCC cell lines SCC-9 and SCC-25 was examined by western blotting. **b** The invasive ability of each tongue SCC cell line was assessed by the Matrigel invasion assay. After 12 h of incubation, invaded cells were fixed and stained with crystal violet. The numbers of cells in six distinct regions of a single chamber were counted. Data are shown as means ± SEM. ***P < 0.001. **c** SCC-9 cells were transfected with two different sequences of siRNAs against *EPB41L5*, and then the Matrigel invasion assay was performed. Data are means ± SEM. *P < 0.05. **d** Cell viabilities of SCC-9 cells with or without the silencing of EPB41L5 was assessed following the exposure to cisplatin and/or radiation. Cisplatin concentrations and radiation doses are as indicated. Data are shown as means ± SEM. ***P < 0.001

We then examined cultured cell lines, and found that SCC-9 cells clearly express EPB41L5 whereas SCC-25 cells do not (Fig. 2a). Both of these cell lines are negative for HPV16/18. SCC-9 cells originated from a tongue carcinoma of a patient with cancer stage T2N1 (‘M’ was not described), and similarly, SCC-25 cells also originated from a tongue carcinoma of a patient with cancer stage T1N1M0 [19]. SCC-9 cells, but not SCC-25 cells, exhibited significant Matrigel invasion activity in vitro (Fig. 2b). siRNA-mediated silencing of *EPB41L5* significantly, but only partially, inhibited the Matrigel invasion of SCC-9 cells (Fig. 2c). Silencing of *EPB41L5* did not notably affect cell morphology (data not shown). These results suggested that the EPB41L5 pathway and the ARF6-based pathway might not be the only pathways driving the invasiveness of SCC-9 cells.

We have previously shown that silencing of *EPB41L5* in other types of cancers, such as breast cancer and renal cancer, drastically improves their drug-resistance [16, 18]. We hence examined whether the same is the case with SCC-9 cells. Contrary to our expectation, however, silencing of *EPB41L5* did not at all improve sensitivity of SCC-9 cells towards cisplatin (50 nM for 72 h) (Fig. 2d). This silencing of SCC-9 cells neither improved sensitivity for ionizing radiation (4 Gy: 2 Gy x 2 times) (Fig. 2d). Anti-cancer drugs are often used in combination with radiation to be effective treatments for HNSCCs [2, 3]. Death rates only by about 20% could be observed with SCC-9 cells upon treatment by cisplatin and radiation in combination, even if their *EPB41L5* was silenced (Fig. 2d). Thus, similar to the above results, these results indicate that although the EPB41L5-based mesenchymal property might in some way participate in the therapeutic resistance of tongue SCC cells, its contribution is very limited, unlike in the case of breast cancer and renal cancer.

**Fig. 3:**
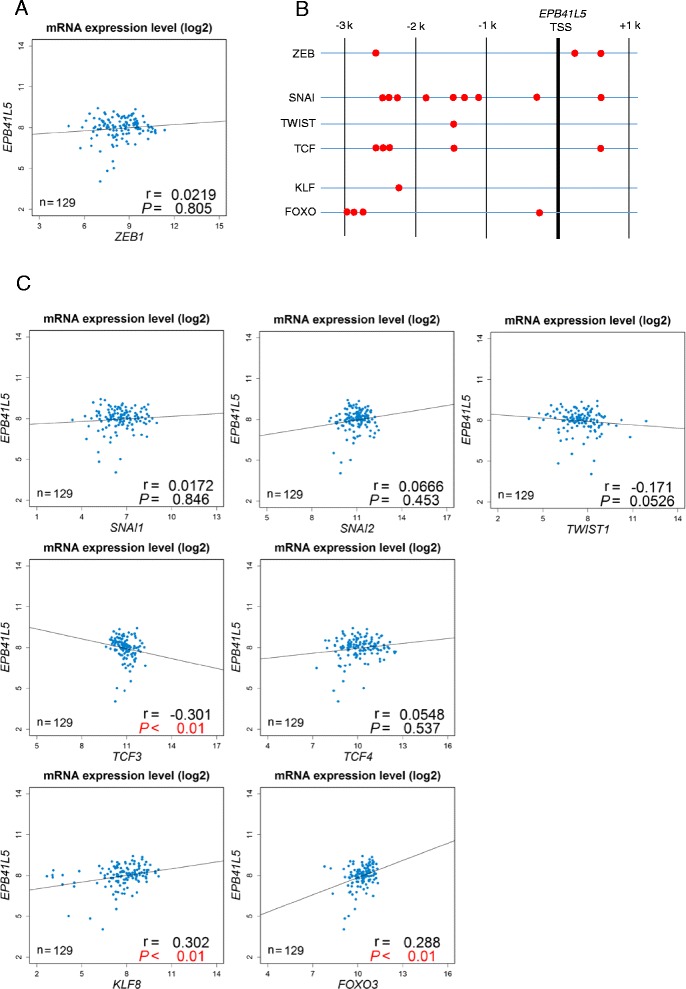
*In silico* search for EMT-TFs that upregulate *EPB41L5*. a Scatter plot of ZEB1-EPB41L5 expression levels. Data were obtained from TCGA RNA-Seq datasets. b Putative binding sites of the indicated transcription factors at the *EPB41L5* locus. JASPAR databases were used. c Scatter plots of mRNA expression levels of the indicated EMT-TFs and EPB41L5. All expression data were converted to log2 values before analysis. To calculate *P*-values, the Spearman rank correlation test was performed

Among the different transcriptional factors driving cancer EMT (i.e., EMT-TFs), ZEB1 has been found to be crucial for the induction of cancer stem cell-like properties and also for the therapeutic resistance of different cancers [4]. Consistently, we have shown that ZEB1 is central in inducing *EPB41L5* in breast cancer cells and renal cancer cells [16, 18]. However, analysis of The Cancer Genome Atlas (TCGA) RNA-Seq datasets on human primary tongue SCCs showed that *ZEB1* mRNA levels do not statistically correlate with *EPB41L5* mRNA levels (Fig. 3a), whereas a tight correlation between *ZEB1* mRNA levels and *EPB41L5* mRNA levels was observed in our TCGA RNA-Seq analysis on human primary breast cancers [18]. In addition to the ZEB1 binding sites, the promoter region of the *EPB41L5* gene contains putative binding sites for the other EMT-TFs, including SNAIL and TWIST (http://jaspar.genereg.net/; see also Fig. 3b). However, the levels of these well-known EMT-TFs also did not exhibit any statistical correlation with *EPB41L5* levels (Fig. 3c). On the other hand, we found that *KLF8* and *FOXO3* show positive correlations with *EPB41L5* with statistical significance (Fig. 3c). However, we were unable to demonstrate that the siRNA-mediated silencing of *KLF8* or *FOXO3*, as well as of *ZEB1*, notably reduces *EPB41L5* expression in SCC-9 cells (data not shown).

In this study, we showed that tongue SCC frequently express EPB41L5, and that its expression levels statistically correlate with the node-positivity and poor outcome of patients. We previously showed that EPB41L5 is the key molecule that drives mesenchymal malignancies in significant populations of breast cancer patients and renal cancer patients [16, 18]. However, unlike these types of cancers, we were unable to obtain evidence supporting the idea that EPB41L5 is the key molecule that drives the invasion and chemoresistance of tongue SCC. Consistently, database analysis suggested that the EMT-TFs that induce EPB41L5 in tongue SCC appear to be different from those of breast cancer and renal cancer. Given the fact that the high expression of EPB41L5 in primary tongue SCC s tightly correlates with poor outcome of the patient, it is likely that the cancer mesenchymal programs, that lead to the EPB41L5 expression as a result of inducing EMT, is more crucial than the EPB41L5 expression itself in promoting malignancies of tongue SCC. Tongue SCC has a high incidence among the different HNSCCs, and similar protocols are generally used for the treatment of the various HNSCCs. Nevertheless, similar studies on other types of HNSCC await to be performed in order to understanding further the mesenchymal programs driving HNSCC malignancies.

The locally advanced invasiveness of HNSCC appears to be the major cause of treatment resistance. Moreover, although chemotherapies and radiotherapies result in notable cancer-reducing effects in most primary lesions of HNSCC, a significant number of HNSCC relapse within a few years, as mentioned earlier. Such treatment resistance may not be merely due to the robustness of HNSCC cells acquired by their mesenchymal and motile phenotypes, but maybe simply due to the fact that the head and neck are vital for the feeding, breathing, and articulation of individuals, and hence areas that can be physically resected or irradiated are tightly restricted. On the other hand, regions within the head and neck, particularly the tongue, are consistently exposed to physical tension as well as many different chemicals from the intake of food and air. Thus, it is possible that epithelial cells of these organs, as well as HNSCC cells in general, may have an innate mechanism to be highly tolerant to such stresses. We found that SCC-9 cells did not die under intensive radiation or a platinum-based drug treatment, which is an unusual property and is unlike the other malignant cancer cells we have examined so far. Thus, the molecular mechanisms as to how HNSCC, such as SCC-9 cells, can tolerate such harsh conditions of genotoxic stress, even in the absence of EPB41L5, deserve further experimental scrutiny, towards achieving a more precise understanding of the molecular profiles of the treatment-resistance of HNSCC.

## Methods

### Cells

SCC-9 and SCC-25 cells were purchased from the American Type Culture Collection and cultured under 5% CO_2_ at 37 °C in Dulbecco’s modified Eagle’s medium/F12 medium (1:1), supplemented with 10% fetal calf serum and 400 ng/mL of hydrocortisone. Antibiotics were not used in our cell cultures.

### RNA interference

siRNA-mediated gene silencing was performed as described previously [6, 10]. In brief, 2 × 10^5^ cells were transfected with 48 nM siRNAs using RNAi MAX kit (Invitrogen), and incubated for 48 h before being subjected to assays. siRNAs targeting *EPB41L5* were chemically synthesized and purified by Japan BioService. An irrelevant siRNA was purchased from GE Healthcare. The specificity and efficacy of the siRNAs were confirmed previously [17, 18].

### Antibodies and immunoblotting

The mouse monoclonal antibody against β-actin was purchased from a commercial source (EMD Millipore). Rabbit polyclonal antibodies against EPB41L5 were established as described previously [17]. Peroxidase-conjugated donkey antibodies against mouse or rabbit IgGs were purchased from Jackson ImmunoResearch Laboratories, Inc. Immunoblotting analysis was performed as described previously [6] using ECL Western detection reagents (GE Healthcare).

### Invasion assay

Invasion assay was performed using 24-well BD BioCoat Matrigel Invasion Chambers with a pore size of 8 μm (BD Bioscience), as described previously [10]. Briefly, 10^5^ cells were seeded in the upper chamber in 500 μL of medium without serum. The lower chamber was filled with 750 μL of complete medium. After 12 h of incubation, cells were fixed with 4% paraformaldehyde for 30 min, and then stained with crystal violet. The numbers of invaded cells were counted in six different areas for every chamber. More than three independent experiments were performed. The Welch *t*-test was performed for group comparisons. Cell viabilities were analyzed using CellTiter 96 (Promega) in accordance with the manufacturer’s instructions.

### Immunohistochemistry and scoring

Immunohistochemical staining was performed as described previously [8, 10, 15]. Briefly, specimens were fixed with formalin and embedded in paraffin, and then sliced sequentially at a thickness of 3 μm. Samples were deparaffinized with xylene and rehydrated with graded alcohol. After rinsing with Tris-buffered saline, samples were processed for antigen retrieval with Dako EnVision FLEX Target Retrieval Solution (pH 9.0), using Dako PT Link at 97 °C for 20 min according to the manufacturer’s instructions. Samples were incubated with antibodies against EPB41L5 (1:1,000) for 30 min, and the Dako Envision FLEX system was used for visualization. The coloring reaction was performed with 3,3′-Diaminobenzidine (Dojin) for 5 min. Hematoxylin was used as a counterstain. The scoring of immunohistochemical samples was performed as described previously [15] by two different investigators, including one pathologist (K.C.H). The H-score value 33% from the top was chosen as the cutoff point for the high-expression group.

### Chemoradiation resistance assay

Cells (1.5 × 10^4^ each) were seeded onto collagen-coated 48-well dishes and then treated with siRNAs for *EPB41L5*. After a 24 h incubation, cells were treated with 50 nM of cisplatin (Sigma), followed by an X-ray irradiation (MBR-1520R-3 HITACHI, 150 kV with a 0.5 mm aluminum filter. 2Gy x 2 times: 48 and 72 h post cell seeding). After a further 24 h incubation, cell viabilities were assessed by using a calcein solution (Dojin). Each assay was performed at least three times, each in duplicate. The Welch *t*-test was performed for group comparisons.

### Patients and specimens

All clinical specimens used in this study were the same as those described in a previous study [15]. Characteristics of the patients were described in a previous report [15] and in Table 1.

### Survival analysis

Disease-free and overall survival rates were analyzed using the Kaplan-Meier method, and *P*-values were calculated by the logrank test. The starting points of the survival rates were the date of surgical resection. The endpoint of disease-free survival was the date of the first diagnosis of disease progression, relapse, or death due to any cause. The endpoint of overall survival was the date of death or the most recent follow-up.

### TCGA analysis

RNA-Seq data sets on tongue SCC were obtained from The Cancer Genome Atlas (URL: http://cancergenome.nih.gov/, *n* = 129). The Spearman rank correlation test was used to calculate *P*-values.

### Statistical analysis

All statistical analyses were performed by R software (URL: https://www.r-project.org/).

## Abbreviations

EMT: Epithelial-mesenchymal transition
EMT-TF: EMT-transcription factor
HNSCC: Head and neck squamous cell carcinoma
TCGA: The Cancer Genome Atlas
Tongue SCC: Squamous cell carcinoma of the tongue
